# Pultruded FRP Beams with Embedded Fibre Bragg Grating Optical Sensors for Strain Measurement and Failure Detection

**DOI:** 10.3390/s21217019

**Published:** 2021-10-22

**Authors:** Daniel Maldonado-Hurtado, Javier Madrigal, Antonio Penades, Rocío Ruiz, Ana Isabel Crespo, Salvador Sales

**Affiliations:** 1 Photonics Research Labs, ITEAM Research Institute, Universitat Politècnica de València, Camino de Vera s/n, 46022 Valencia, Spain; damalhur@iteam.upv.es (D.M.-H.); jamadmad@iteam.upv.es (J.M.); anpepl2@upv.es (A.P.); 2 Technology Department, AIMPLAS Research Center, Gustave Eiffel, 4, 46980 Paterna, Spain; rruiz@aimplas.es (R.R.); acrespo@aimplas.es (A.I.C.)

**Keywords:** fibre Bragg grating, composite, pultrusion

## Abstract

Composites have added new dimensions to the design and construction of buildings and structures. One of the wider spread composite applications in the construction industry is composite beams or pillars, which can be manufactured through pultrusion processes. These types of construction elements are usually used to withstand the weight of large loads, so their integrity must be guaranteed. Due to optical sensors’ advantages over their electrical counterparts—small sizes, low weight, non-conductive, and immunity to electromagnetic interference—and FBGs having an outstanding position among optical fibre sensors—due to their multiplexation capability and relatively easy monitoring—in this study, we propose the integration of FBG sensors for the observation and analysis of the integrity of structures made with composite beams over time. The validation test results showed the successful embedding integration of FBG-based fibre optical sensors in an FRP pultrusion beam and strain transmission up to 7500 µɛ from the composite test piece to the sensor. Additionally, we were able to anticipate the piece failure by the FBG spectrum deformation.

## 1. Introduction

Composite materials are formed at macroscopic scale from the combination of two or more different materials. This allows the structural use of materials in forms with superior properties to the constituent elements. Within the composite, the difference between materials can be easily recognized as they do not dissolve or blend into each other. Composites exist in nature; a piece of wood, for example, consists of long fibres of cellulose held together by a much weaker substance called lignin. Cellulose can also be found in cotton and linen, but it is the binding power of the lignin as a matrix that makes a piece of timber much stronger than a bundle of cotton fibres [1,2].

Nowadays, composite materials are present in almost every industry in some form or format. Since composite materials can be manufactured into practically any shape, they allow great design flexibility: the selection of the optimum constituents through design and studies, and the ability to tailor them to obtain the properties required by a specific task. Additionally, composites can be chemical- and corrosion-resistant depending on the matrix compound. Furthermore, conventional engineering materials generally have a higher weight and a worse strength-to-weight ratio than composites. These improved ratios can reduce system weight by up to 30%, obtaining better performance and reducing energy consumption in the transport sector. Elevated creep resistance and good damping qualities are some properties reached by advanced composites. Moreover, composites are considered an alternative to metallic components, such as in the airframes, where fatigue loads due to metallic materials can damage the structure [3].

The presence of composite materials in the industry will continue to grow as engineers and academia develop more composite analyses, studies, fabrication systems, constituent materials, and designs. In summary, the acceptance and future of composite materials in the industry will depend on their ability to be tailored, design versatility, and low environmental impact, despite the low processing cost [4].

As a brief review, the transport industry has made extensive use of composite materials. Their light weight, high strength, and ability to be manufactured into complex shapes, such as aerodynamic surfaces, have resulted in lower fuel costs. The corrosion resistance of the composite materials allows a reduction in the maintenance costs and extend the service life of many parts and products: auto and truck bodies and parts, trailers, tanks, special-purpose vehicles, and manufacturing equipment [5]. Moreover, the aerospace and military sectors are at the technological forefront of the improvement and development of composite materials. In these industries, the continuous search for stiffer, stronger, and lighter materials for large-scale structures, together with a large volume of economic resources, allow great opportunities for composite materials to show their superiority over traditional materials. As previously mentioned, durability and low maintenance are additional assets of composite materials. The development of novel manufacturing processes and the optimization of conventional ones have allowed a great reduction in some manufacturing costs; for example, there have been reductions in the number of parts required to construct some components by using composite materials instead of traditional ones such as metals. Furthermore, new military aircrafts almost exclusively use advanced composites for structural components and parts, as rocket engine cases, nozzles, and nose cones are missile applications in which a great number of composite materials are used. Others, e.g., radar domes, rotor blades, propellers, and many secondary structure components, such as fairings, doors, and access panels are also fabricated from advanced composites in the aeronautical world [6]. Even in the sporting goods industry, advanced composites have become the default material for sports equipment, providing performance and safety improvements for participants, relegating the traditional materials to a secondary place. For example, there are composite-based tennis rackets, golf clubs, ski accessories, sailing hydrofoils, diving equipment, and many other sports equipment [7].

On the other hand, composites have added new dimensions to the design and construction of buildings. Their excellent benefits described above mean that composite materials have a significant impact on the industry. In the construction sector, composite materials have reduced the manufacturing cycle time by providing a more flexible design of the structures in civil applications [8]. One of the wider spread composite applications in the construction industry is with composite beams or pillars, which can be manufactured through different composite manufacturing processes (CMPs). These types of construction elements are usually used to withstand the weight of large loads and structures, so their integrity must be guaranteed.

Several ad hoc numerical and experimental studies of thermochemical analysis have been performed to provide a better understanding of the pultrusion process by evaluating the development of temperature and degree of cure profiles within the heating matrix [9,10,11,12,13,14,15,16,17,18]. Some of the literature includes thermo-mechanical studies that focus on the main mechanisms which drive the evolution of the residual stresses, strains, and deformations based on the incorporation of fibre optical sensors (FOS) into composite beams [19,20]. Pressure is another parameter that has been studied by embedding FOS based on acoustic emission or plasmonic resonators [21,22], or even the study and experimentation of automated produced pultruded beams with FOS [23,24]. 

Additionally, some studies have focused on structural health monitoring (SHM) for composite beams [25]. SHM allows damage identification in structural systems. Smart materials are obtained by the integration of sensors in composites that can be permanently placed which, along with decision-making algorithms, can detect structure damage, observe and analyse structural integrity, and predict the remaining lifetime of structures, replacing or displacing the traditional non-destructive evaluation (NDE) for confirmation of damage detection. 

However, the integration of optical sensors for observation and analysis of the integrity of structures made with composite beams over time has not been considered until now. Optical fibre sensors have been widely used for Fibre-Reinforced Polymer (FRP) composite monitoring in recent last years, due to their advantages over their electrical counterparts, small sizes, low weight, non-conductivity, and immunity to electromagnetic interference. These properties, among others, make optical fibre sensors suitable for embedding into FRP applications, such as energy, aerospace, automotive, and structural health monitoring [26,27]. 

Fibre Bragg gratings (FBGs) have an outstanding position among optical fibre sensors, due to their multiplexation capability and relatively easy monitoring [28,29], including flexibility for wavelength shift determination or damage detection by post-processing signals in the case of reflected spectrum modification by nonuniform strain distribution in the piece. 

In this paper, we discuss the successful embedding integration of FBG-based fibre optical sensors in an FRP pultrusion beam and prove sensor adherence and strain transmission up to 7500 µɛ from the composite test piece to the sensor. Additionally, we were able to anticipate the piece failure by the FBG spectrum deformation.

The authors have worked in several environments, using FBG sensors for SHM of beam structures. As seen in [30], several FBG sensors were installed in one face of a stainless-steel cantilever beam to test their performance for a modal analysis of vibrating beams, both in air and partially submerged in water, studying its applicability in the hydropower industry. Some limitations found in this study were the fragility of the sensors of extremely small diameters and the fact that their installation requires trained staff. However, this is the first time that the authors have embedded FBG sensors directly into the production line of a pultruded FRP beam to study their performance and applicability for SHM. The overarching functionality obtained for strain measurement, failure detection, and the additional protection intrinsic of the embedding process for FOS allows the implementation of smart FRP pultruded components into the industry, overcoming the limitations described before.

## 2. Materials and Methods

### 2.1. FRP Beam Fabrication

FRP beams can be produced by several CMPs other than pultrusion, such as autoclave, vacuum infusion, and resin transfer moulding (RTM). Nevertheless, if we compare all these processes, pultrusion is the unique continuous manufacturing process resulting in the steady output of a composite profile, which is cut to length at the end of the process. Thus, in this work, it was used for beam manufacturing. During the pultrusion process, roving continuous fibres are pulled through a resin bath where they become impregnated with resin and are later pulled through a heated die. Once the impregnated fibres enter the heated die, the cross-linking process of the resin begins; as they are pulled through the die, the resin gradually polymerizes and the fibres and resin copy the shape of the given die. It is extremely important to maintain an optimal temperature gradient inside the die as any alteration will affect the quality of the final composite. The beam exits the die once the resin is cured. Subsequently, the composite beam will be cut to the desired length by a saw system at the end of the process line. 

**Figure 1:**
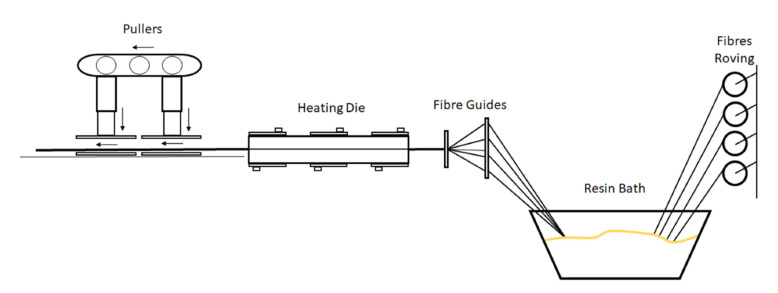
FRP pultrusion process used.

To reach the optimum cross-linking temperature, the heating die has several thermal resistances parametrized according to the resin characteristics. The pulling speed must be settled according to the gradient of temperatures introduced in the heated die. The optimum parameters for the best cross-linking will depend on the pulling speed and the temperature gradient inside the die. The selection of the pulling speed and temperature gradient allows for stabilization within the manufacturing process, avoiding the overheating of the piece, as this can cause resin degradation and, therefore, the jamming of the beam inside the heated die [19]. A scheme of the fabrication process can be seen in Figure 1.

### 2.2. Fibre Optic Embedded Integration

**Figure 2:**
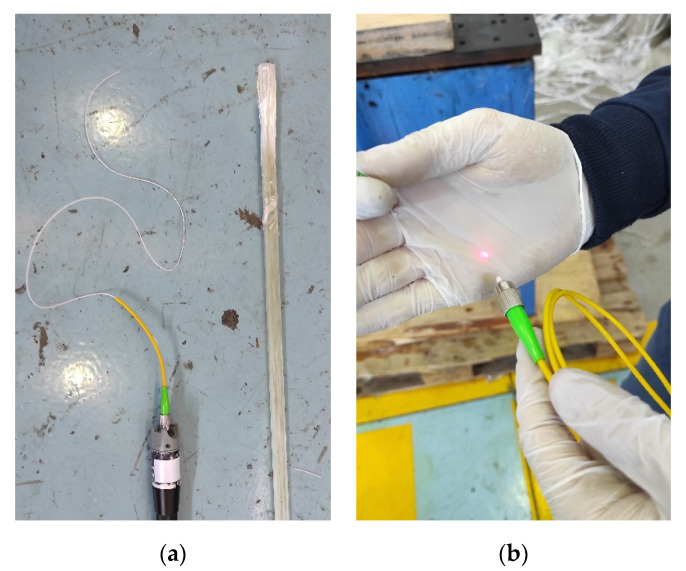
Single mode fibre embedded integration test: (**a**) fibre fault detector connexion on one embedded fibre end; (**b**) embedding survival test with light exiting the SMF on the other end of the embedded fibre.

The integration of standard single mode optical fibre (SMF) in the direction of the reinforcement (glass fibre threads in this case) was decided in this first experimental stage. Thus, standard SMF was embedded in the centre of the pultruded beam. The optical fibre survived the whole process, tested through the connexion of a fibre fault detector laser at one end of the beam and checking how the light is able to travel into the fibre until the opposite end of the beam is reached, as seen in Figure 2a,b, respectively.

### 2.3. Beam Testing

**Figure 3:**
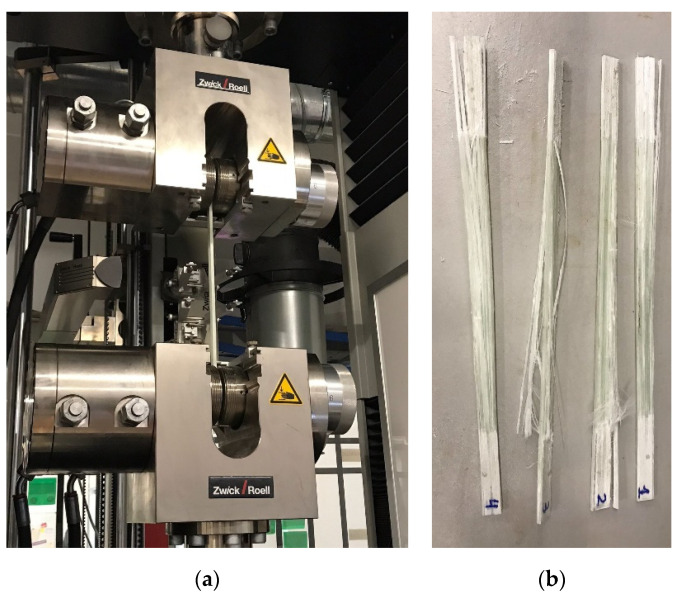
Beam testing scheme: (**a**) tensile test machine beam configuration; (**b**) beam test pieces with optical embedded fibre tested to failure on the machine.

A tensile test machine Zwick/Roell AllroundLine Z250 provides the material testing equipment and software to complete the tensile test on the beams. To measure the actual deformation of the test piece, a longitudinal extensometer MakroXtens was located on the central part of the tested piece with an initial distance of 50 mm between points. The tested piece was held on both sides with hydraulic grips designated for composites testing, as shown in Figure 3a.

Four beam test pieces with embedded optical fibres were manufactured to test the possible effect that embedded optical fibres can have on the overall strength of the beam, carrying out mechanical tensile testing until failure, as seen in Figure 3b; we obtained the results shown in Table 1, where the average deformation at tensile strength result was 1.42%.

According to the testing provided by AIMPLAS on similar beams on 22-Oct-2020 (UNE-EN ISO 527-4) shown in Table 2, the related deformation at tensile strength was 1.44%. In this regard, we can conclude that the integration of embedded fibres did not affect the mechanical axial strength of the beam.

### 2.4. FBG Strain Sensing Principle

Fibre Bragg Gratings produce a narrow-band reflection of incident light that satisfies grating reflection conditions [31]. The basic principle is:

(1) $$\lambda =2n_{eff}\Lambda$$

*λ* is the reflection peak wavelength, also known as the Bragg wavelength; *Λ* is the grating period; *n_eff_* is the effective refractive index. Due to stretching of the grating period, the grating has a wavelength change when FBG is subjected to an axial strain [32]. Assuming that the sensor is only subjected to tensile strain ɛ and under constant temperature, the axial strain can be expressed as:

(2) $$\varepsilon_{\mathrm{xx}}=\frac{\Delta \lambda }{\lambda \cdot K_{e}}$$

where K_e_ is the strain-optic coefficient of the standard SMF optical fibre (0.78 × 10^−^^6^) and *λ* is the Bragg wavelength. From Equation (2), we can see that the Bragg wavelength shift and the longitudinal strain have a linear relationship at constant temperature. The equation can be simplified to:

(3) $$\Delta \lambda \left(\varepsilon \right)=S_{\varepsilon }\cdot \varepsilon_{\mathrm{xx}}$$

where S*_ɛ_* is the FBG strain sensitivity and with an approximate value of 1.2 pm/μɛ, which is somewhat dependent on the dopant species and concentration in the core of the fibre, but also in a lesser extent to the composition of the cladding and coating, variations of 5–10% are usual between standard optical fibres [33]. As seen in Equations (2) and (3), S*_ɛ_* is also related to Bragg wavelength. In this case, as the sensors are fabricated in the C-band window, *λ* has been considered a constant value of 1550 nm. 

The influence of strain on the beam causes the Bragg wavelength shift of the FBG sensor; in this way, the FBG sensor can be used to monitor the longitudinal strain load on the beam.

### 2.5. FBG Sensors Integration

**Figure 4:**
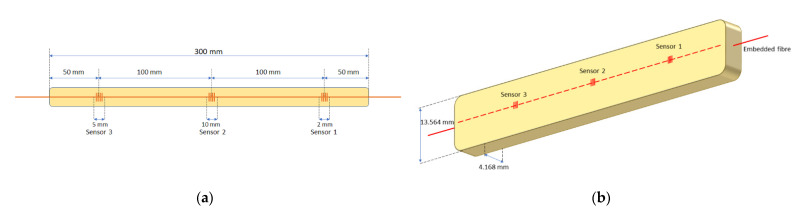
FBG sensor configuration: (**a**) ubication scheme and naming of sensors; (**b**) scheme of beam fabrication, with embedded optical fibre inside.

**Figure 5:**
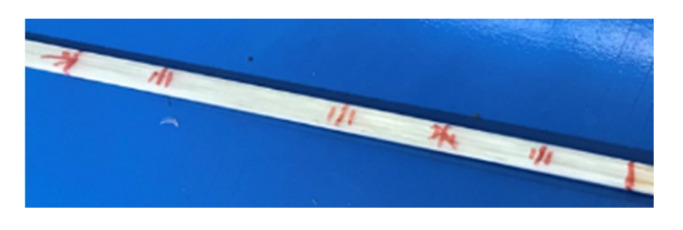
Sensorized FRP beam test piece.

Once the optical fibre embedding process was fully defined, we continued with the integration of the optical fibre sensors. To assess the technical feasibility of the proposed technique, three FBG sensors of different lengths, 2 mm, 5 mm, and 10 mm, and different Bragg wavelengths, 1558.0 nm, 1548.5 nm and 1553.3 nm, were used, respectively, so that they could be multiplexed. The FBG sensors were manufactured using the well-known phase mask method using a continuous wave doubled frequency 244 nm argon ion laser to inscribe the gratings in a standard single mode fibre [31]. In Figure 4, an FBG sensor’s naming and position scheme is shown. In this way, a sensorized beam test piece was obtained, as shown in Figure 5.

Strain sensor measurement was performed through the detection of the Bragg wavelength change in the reflected spectrum of each FBG sensor. An optical sensor interrogator device, “Micron Optics sm125”, was used for the FBG spectrum acquisition. The interrogator employed a full spectrum scanning from 1510 nm to 1590 nm through the Micron Optics patented Fibre Fabry-Perot Tunable Filter technology. It also includes 4 independent time-multiplexed channels and a 2 Hz scan frequency [34]. The strain coefficient used to convert the wavelength change to strain was 1.22 pm/µɛ.

## 3. Results

### 3.1. Embedded FBG Sensors Strain Measurement

**Figure 6:**
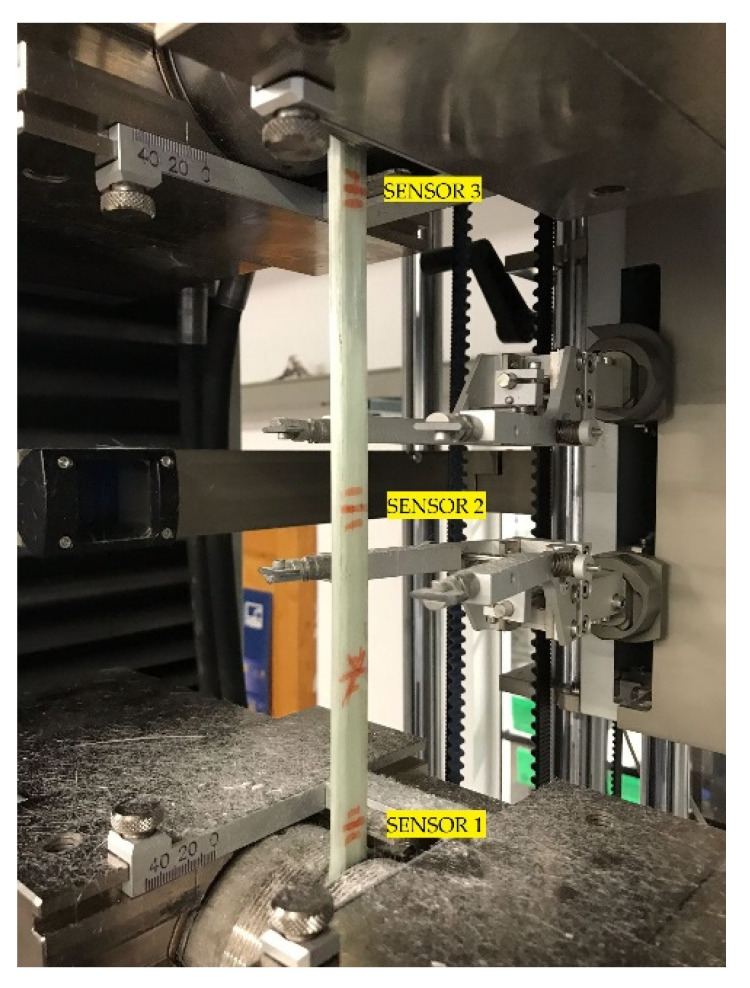
Sensorized beam test piece setting.

A mechanical tensile cycle test on the sensorized beam test piece was carried on studying the answer given by the embedded optical fibre sensors. Based on the results obtained in the beam testing section, we defined the intervals of deformation for the tensile cycles. As the average deformation of the beam before failing was 1.42%, the lower limit, in all cases, was stablished at 0.25% of deformation, and as an upper limit, 0.50% and 0.75% of deformation was settled. For each study case, the tensile cycles strained the beam between the lower and the upper limit ten times, at a constant speed of 2 mm/min. In Figure 6, the layout of the test piece on the tensile testing machine and the position of the sensors can be seen.

As is known, FBG sensors are principally sensitive to strain and temperature [31]. In this controlled experimental setup, to obtain FBG strain measurement, the general temperature of the environment was kept constant throughout all the cycles. In [35,36,37], several strain–temperature decoupling techniques have been used in uncontrolled environments.

**Figure 7:**
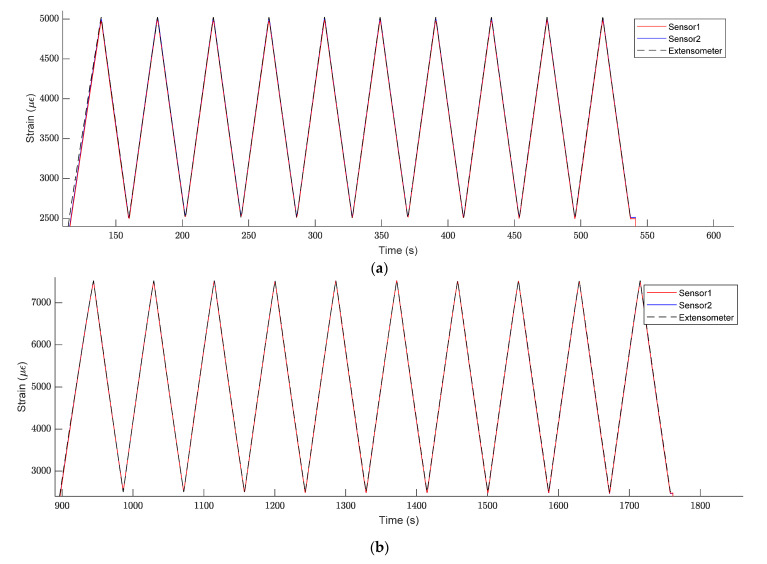
Tensile strain cycles: (**a**) 0.25–0.50% strain cycles; (**b**) 0.25–0.75% strain cycles.

The strain measured by the FBG sensors in both tensile cycles can be seen in Figure 7a,b. Sensors 1 and 2 show the same readings throughout the cycles. Additionally, the strain measured by FBG sensors had a close match with the strain measured by the longitudinal extensometer located on the central part of the tested piece, giving us the following important information:

- As sensors survived and information was useful, the procedure used to embed a standard SMF optical fibre with FBG sensors during FRP pultrusion for sensorized beam manufacturing was successful.
- The standard SMF shows enough adherence to transfer strain from the piece to the fibre sensor. No slippery was detected in any cycle.
- This embedding of optical sensors in the part itself in its manufacturing process provides great benefits, such as insulation of the sensors against external agents since they are found inside the beam. Additionally, the FBG sensor built in a standard optical fibre was able to read a strain of up to 7500 µɛ, which is 65% higher than the normal guaranteed strain reading before slippering of that kind of optical fibre [38].

### 3.2. Beam Failure Detection

**Figure 8:**
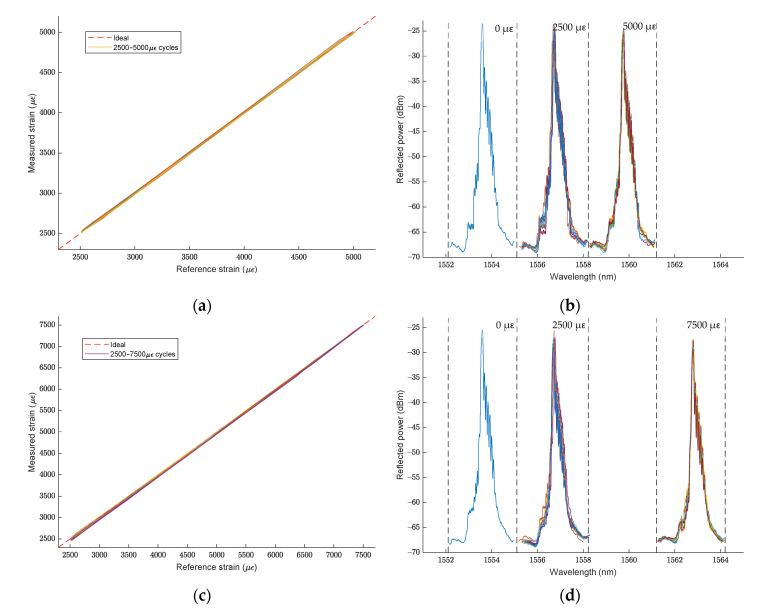
Sensor 2 strain readings: (**a**) accuracy at 0.25–0.50% strain cycles; (**b**) FBG spectrum conservation at 0.25–0.50% strain cycles; (**c**) accuracy at 0.25–0.75% strain cycles; (**d**) FBG spectrum conservation at 0.25–0.75% strain cycles.

Figure 8 shows the strain accuracy of Sensor 2 compared with the strain measured by the longitudinal extensometer and the spectrum of the FBG sensor on the tensile cycles ran over the FRP sensorized beam, for the 0.25–0.50% strain cycles (a) and (b), and the 0.25–0.75% strain cycles (c) and (d). The segmented red line in (a) and (c) represents the ideal relationship between measured and reference strain; as can be seen, there is a close correlation between them. The spectrum of the FBG sensor shown in (b) and (d) is the overlap of the recorded spectrum in the extreme points of the tensile cycles, which are 0 µɛ, 2500 µɛ, 5000 µɛ and 7500 µɛ, respectively.

As seen in previous sections, embedded FBG sensors accurately monitor the tensile strain loaded onto the beam over the sensorized point, over several tensile cycles, as seen in Figure 8a,c. Monitoring the datum determines that its safety has been compromised if the structure has been subjected to a strain that exceeds its safe working load limit. The FBG strain sensing principle is to follow the Bragg wavelength change to determine the strain change; if the strain distribution in the beam is homogeneous, there will be no noticeable changes in the FBG spectrum, as can be seen in Figure 8b,d.

**Figure 9:**
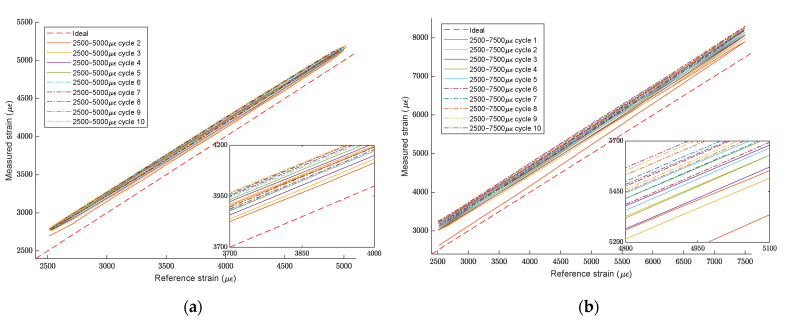
Sensor 3 strain readings: (**a**) accuracy at 0.25–0.50% strain cycles; (**b**) accuracy at 0.25–0.75% strain cycles.

Sensor 3, located on the top of the beam, as shown in Figure 6, shows a bigger strain than the other two sensors. The tensile test machine cannot detect this anomaly as it measures and controls the strain from the extensometer installed in the centre of the test piece, where Sensor 2 shows a regular strain change between each cycle, as defined before. In Figure 9, the strain accuracy of Sensor 3 compared with the strain measured by the longitudinal extensometer is shown.

As seen in Figure 9a, even if readings of Sensor 3 at the 0.25–0.50% strain cycles are far from the ideal relationship between the measured and reference strain, they stay similar between cycles and have a consistent slope related to the ideal line. However, in Figure 9b, the step-up part of the first 0.25–0.75% strain cycle shown in the red continuous line exhibits a big slope increment in the relationship studied. Afterwards, readings of Sensor 3 at the 0.25–0.75% strain cycles continue increasing with every iteration.

**Figure 10:**
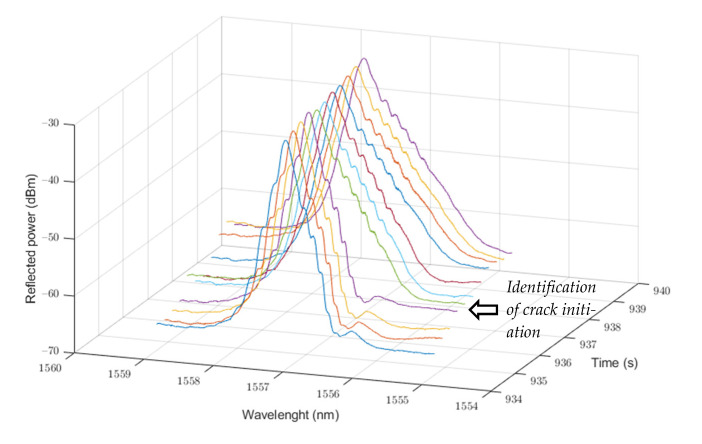
Sensor 3 FBG spectrum change at first 0.25–0.75% strain cycle.

In Figure 10, the study of the FBG spectrum of Sensor 3 in the step-up part of the first 0.25–0.75% strain cycle reveals a visible change in spectrum at 938 seconds. This sudden change in the FBG spectrum shape is produced by the non-homogeneous strain distribution over the extension of the FBG and has been used in several studies for the delamination or crack identification in FRP composite structures [39,40].

The successive strain increment in the top part of the test piece induced an anticipated failure of the tested part; after ending the second cycle test and releasing the tension from the beam, the test piece cracked and failed. Thus, embedded optical fibre FBG sensors can be used to anticipate an FRP beam structure failure that indicates an imminent structure end of life.

## 4. Discussion

The embedding of standard SMF optical fibre FBG sensors during the fabrication of FRP pultruded beams was successfully completed and enabled the fabrication of smart FRP beams that can be used to implement SHM in structures. All sensors survived the embedding procedure, and no unusual behaviour was detected during the process.

Standard SMF shows enough adherence to transfer strain from the piece to the fibre sensor. No slippery was detected on installed sensors. 

The embedding of optical sensors in the part itself in its manufacturing process provides great benefits, such as insulation of the sensors against external agents since they are found inside the beam. Furthermore, the FBG sensor built in a standard optical fibre was able to read a strain up to 7500 µɛ, which is 65% higher than a normal guaranteed strain reading before slippering of that kind of optical fibre.

Embedded optical fibre FBG sensors can be used to anticipate an FRP beam structure failure that triggers a structure replacement, as they can detect strain load or even detect small delamination or disengagement between matrix and fibres through FBG spectrum change.

The different lengths of the FBG sensors do not affect their accuracy for strain reading. Nevertheless, a larger length of the FBG sensor could lead to an increment in sensor sensitivity to piece crack, as the non-homogeneous distribution of the strain over the extension of the sensor would notoriously change the FBG spectrum shape.

## Figures and Tables

**Table 1:** Tensile testing results of test pieces with embedded optical fibre.

Series	Thickness	Width	Modulus of Elasticity	Tensile Strength	Deformation at Tensile Strength
n = 4	mm	mm	MPa	MPa	%
Mean value	4.168	13.564	52,900	740	1.42
Standard deviation	0.045	0.069	2360	22.3	0.0263

**Table 2:** Tensile testing results of resin with glass fibre cured PRO18-0264-01-00-01.

Series	Thickness	Width	Modulus of Elasticity	Tensile Strength	Deformation at Tensile Strength
n = 5	mm	mm	MPa	MPa	%
Mean value	4.958	14.805	38,400	584	1.44
Standard deviation	0.141	0.075	1900	27	0.14
